# Two decades of dental malpractice litigations in Türkiye: a retrospective matched cohort study analyzing legal and clinical outcomes

**DOI:** 10.1186/s12903-025-05838-1

**Published:** 2025-04-04

**Authors:** Esra Yuce, Seymanur Kacdıoglu Yurt

**Affiliations:** 1 Department of Oral and Maxillofacial Surgery, Faculty of Dentistry, İstanbul Aydın University, Istanbul, Türkiye; 2 Private Clinic, Oakville, ON Canada

**Keywords:** Malpractice, Forensic dentistry, Jurisprudence, Negligence, Litigation

## Abstract

**Background:**

This methodological study aims to provide a comprehensive database of dental malpractice cases in Türkiye over the past 20 years, with a focus on the patterns of malpractice claims across different specialties and settings, as well as the characteristics of the events that give rise to litigation. The study also seeks to clarify to raise awareness of patient safety among dental practitioners to enhance care quality and liability risk management by providing insights into the legal outcomes of malpractice cases.

**Methods:**

A total of 100 dental malpractice claims spanning 23 years (2000–2023) were included in this retrospective, matched cohort study. The cases were categorized into four groups: Malpractice; Complication; Undetermined; and Unresolved. The analysis focused on various legal and clinical variables, including the type of dental treatment, the physician’s level of duty, the presence of auxiliary healthcare personnel, the type of healthcare institution, the legal outcome of the case (decision, settlement, and compensation status), the reasons for filing the malpractice claim, and the appointment of expert witnesses. Data were analyzed using the chi-square test and Fisher’s Exact Test, with statistical significance set at *p* < 0.05.

**Result:**

The majority of cases were related to prosthodontics (31%) and oral surgery (24%), followed by oral diagnosis (14%), implantology (12%), orthodontics (9%), endodontics (5%), restorative dentistry (2%), pedodontics (2%), and periodontology (1%). The most common reason for malpractice claims was incorrect treatment (88%), followed by incomplete treatment (33%), misdiagnosis (32%), patient fault (21%), treatment delays (19%), lack of follow-up (16%), failure to obtain informed consent (10%), delays in diagnosis (3%), document forgery (3%), and infectious disease (2%).

**Conclusion:**

This study highlights the importance of thorough planning, assessments, and preventive measures in dental practice, particularly in prosthodontics, oral surgery, and implantology, which involve invasive procedures, prolonged treatments, and high costs—factors that contribute to higher patient dissatisfaction and increased malpractice risks. Addressing these factors through improved oversight and decision-making could reduce the frequency of litigation and minimize legal disputes.

## Clinical trial number

Not applicable.

## Introduction

Since the emergence of medical science, the primary objective of healthcare has been the restoration of patient health, with patient safety becoming a central focus across all healthcare professions [1, 2]. In this context, the World Health Organization (WHO) and nearly all healthcare organizations have prioritized patient safety which aims to prevent avoidable adverse events, such as accidents, errors, and complications, particularly in dentistry, while also minimizing the impact of unavoidable adverse events [2]. However, in recent years, this fundamental goal has been complicated by the increasing influence of legal principles and sanctions, particularly through defensive behavior in medical practice aimed at shielding physicians from liability rather than prioritizing patient care which has imposed unnecessary costs on patients and the healthcare system, negatively impacted patient access to care, and prolonged medical treatment processes [3]. One of the key factors contributing to this shift is the widespread confusion among patients and their families regarding the distinction between medical malpractice and adverse event [4]. The individual’s integrity is examined within the context of medicolegal standards and assessed based on the specific guidelines set forth in each country [5]. However, it is widely accepted that if adverse event is caused by a failure to meet the standard of care, it is considered a physician error [6]. Treatment failure, distinct from medical error, may not be due to the physician’s fault, but rather to factors such as the type/character, nature, and stages of the disease, the actual state of available effective treatment options, comorbidities, and the patient’s vital strength [7]. The distinction is made between whether the adverse outcome resulted from failure to adhere to proper procedures, negligence, or error in which case it would be considered an error or whether it is an inevitable, natural risk of the treatment, in which case it would be considered a complication [8]. Negligence is a form of error that is difficult to justify, resulting from a lack of knowledge or basic skills, failure to take minimal precautions, or neglect. In contrast, a complication is an unfavorable, unintended, but often unavoidable, negative outcome that arises from a procedure or treatment, even when proper care is provided [9]. Risk management in healthcare is interpreted as a process aimed at identifying, monitoring, assessing, and mitigating threats that may potentially affect patient safety; this includes both preventable errors and unavoidable complications [10]. At this point, medicolegal evaluation seeks to collect data that connects an iatrogenic traumatic procedure to the harm suffered by the patient, requiring a clear causal link between the physician’s actions and the injury to establish negligence in medicolegal cases [5].

Medical malpractice is based on several foundations including intentional wrongdoing, breach of contract, defamation, disclosure of confidential information, insufficiently informed consent, failure to prevent foreseeable injuries, and medical negligence with the latter being the dominant theory underlying most malpractice cases [2, 5, 8, 11]. It is also essential to note that the responsibility for medical malpractice extends beyond individual physicians. Healthcare institutions have an obligation to ensure that care is provided in compliance with medical standards and regulations. Identifying fault in malpractice cases can be difficult due to the involvement of multiple parties, including the healthcare institution, medical staff, and external factors such as equipment failure [2].

In dental practice, negligence is the most prevalent form of liability, with unexpected complications or adverse outcomes, such as implant failure, inferior alveolar nerve damage, maxillary sinus involvement, and injury to adjacent teeth, frequently leading to malpractice disputes in the dental and oral-maxillofacial field [2, 11]. The growing attention to malpractice and professional liability in modern dental practice highlights the increasing significance of adopting strategies that integrate both risk management and patient safety, focusing on identifying and mitigating potential issues that could harm patients, avoid malpractice claims, and offers legal protection or dental practitioners, all of which ensure the delivery of safe and high-quality care [12]. In this manner, analyzing the actions that lead to lawsuits against dental practices, as well as the legal and medical aspects of such cases, enhances our understanding of current situation that need improvement and attention, ultimately ensuring patient safety [13, 14].

Despite the increasing number of malpractice claims globally [15], there is a lack of comprehensive, quantitative analysis regarding resolved dental malpractice cases in Türkiye. The main objectives of this study were to assess the most common dental specialties involved in malpractice cases, evaluate the legal outcomes of these cases, and the factors that contributed to their occurrence. Our second goal is raising awareness and familiarity with patient safety among all dental practitioners aimed at improving patient safety, quality of care, and liability risks management by providing resources and the legal outcomes of these cases.

## Materials and methods

### Study design

This study was designed as a retrospective, matched cohort study to analyze dental malpractice cases in Türkiye between 2000 and 2023. A total of 8140 dental malpractice cases were initially identified, including final as well as pending decisions by the Supreme Court of Appeals of the Republic of Türkiye. All cases were obtained from the Supreme Court of Appeals IT Directorate database (karararama.yargitay.gov.tr) using the keywords: “Malpractice in Dentistry”, “Malpractice Cases in Dentistry”, “Dentist”, “Prosthodontics”, “Oral Surgery”, “Implantology”, “Endodontics’’, “Periodontology”, “Pedodontics”, “Restorative Dentistry”.

This study adhered to the Declaration of Helsinki on medical ethics and has been approved by the Acıbadem University Human Research Ethics Committee (Approval No: 2023-07/239). In accordance with national regulations, the requirement for informed consent was waived by the ethics committee, as the data in the Supreme Court of Appeals IT Directorate database were anonymized.

### Data collection

The study included cases filed against dentists and/or healthcare institutions for malpractice in dentistry. Inclusion criteria were as follows:

- Cases involving individuals legally practicing dentistry under Article 41 of Law No. 1219 of the Turkish Constitution.
- Lawsuits arising from oral and dental health examinations and treatments.
- Cases where the physician personally conducted the diagnosis and treatment of the patient, and the patient or their relative filed the lawsuit.
- Cases with complete and accessible dental and legal records.

Cases were excluded from the study based on the following criteria:

- Non-malpractice cases or those filed for reasons other than malpractice.
- Defendants who were practicing dentistry illegally (i.e., without a valid dental degree, as per Article 41 of Law No. 1219).
- Lawsuits related to issues outside of oral and dental health examinations and treatments.
- Cases where the physician did not directly carry out the diagnosis and treatment of the patient.
- Duplicate cases identified by the data provider.

A total of 8140 cases were identified through the systematic search. After removing duplicates, all results were initially screened based on the content of the case to assess their relevance. The second level of filtering evaluated the cases’ eligibility for inclusion and exclusion based on predefined criteria. Finally, a third level of filtering was applied to assess the availability of data. Of the initial cases, 6284 were excluded based on exclusion criteria (e.g., duplicate cases, non-malpractice cases), 1,020 did not meet the inclusion criteria (e.g., incomplete legal or dental records, defendants practicing without a license), and 296 had missing data (e.g., unidentifiable adverse events, missing dates or causes). Ultimately, 100 cases met the inclusion criteria and were selected for analysis. All screenings were conducted independently by one author (SK) and subsequently re-checked by another author (EY).

**Fig. 1:**
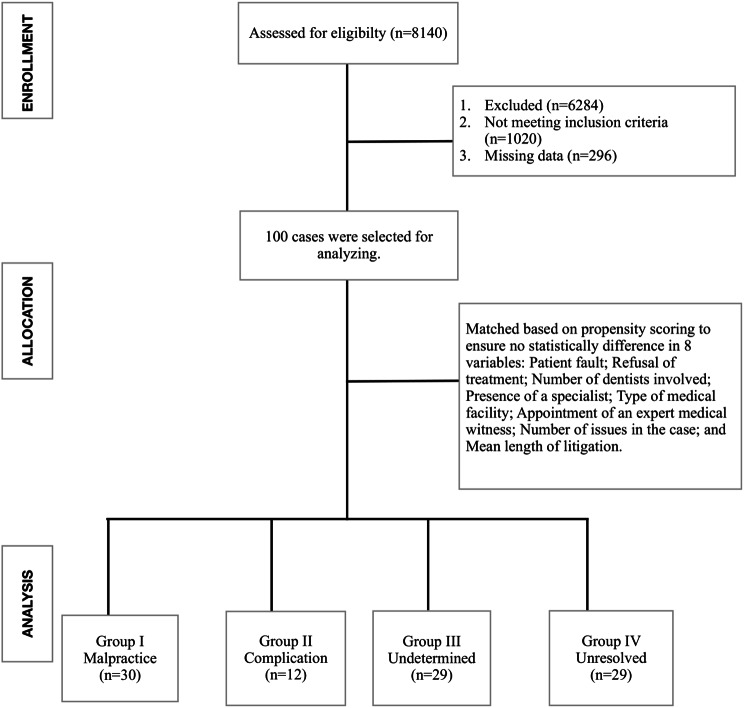
Flowchart of the sample selection

A matched analysis using propensity score matching was performed to control for confounding variables, based on eight key factors: Patient fault; Refusal of treatment; Number of dentists involved; Presence of a specialist; Type of medical facility; Appointment of an expert medical witness; Number of issues in the case; and Mean length of litigation (Fig. 1).

These 100 cases were subsequently divided into four groups based on the final legal decision regarding the presence of malpractice:

- Group I (Malpractice): 30 cases where malpractice was confirmed.
- Group II (Complication): 12 cases where the outcome was classified as a complication.
- Group III (Undetermined): 29 cases where it was not possible to distinguish between malpractice and complication.
- Group IV (Unresolved Decision): 29 cases where the decision was inconclusive, as they were appealed to higher courts and returned for further action due to deficiencies in the case files, such as incomplete investigations, insufficient documentation, or failure to hear all witnesses, among others.

### Data variables and assessment

Legal and clinical data were extracted from the court case files, which included the following variables:

- Clinical: Type of dental examination and treatment applied (within the scope of the nine dental field: oral surgery, implantology, periodontology, orthodontics, prosthodontics, endodontics, pedodontics, oral diagnosis and radiology, and restorative dentistry), physician’s duty level, presence of auxiliary healthcare personnel, and type of healthcare institution.
- Legal: Case decision status, settlement status, compensation status, reasons for filing the malpractice claim, and appointment of expert witnesses.

### Statistical analysis

Data were analyzed using IBM SPSS Statistics 22 (IBM SPSS, Türkiye). A power analysis was performed using G Power software (version 3.1.9.2). With a power of 0.80, significance level set at *p* < 0.05, and effect size (d) of 1.051, the minimum required sample size was calculated to be 12 per group.

Descriptive statistics (frequencies) were used to summarize the data. The Chi-square test and Fisher’s Exact Test were applied to compare categorical variables. Statistical significance was considered at *p* < 0.05.

## Results

This study analyzed 100 dental malpractice claims over a 23-year period (2000–2023). The cases were categorized into four groups: Group I (malpractice, *n* = 30), Group II (complications, *n* = 12), Group III (undetermined, *n* = 29), and Group IV (unresolved, *n* = 29). The groups were matched based on propensity scoring, and no statistically significant differences were observed in the matching variables. Expert witnesses were consulted in 59% of the cases, with only one case reaching a settlement.

The majority of cases were related to prosthodontics (31%) and oral surgery (24%), followed by oral diagnosis (14%), implantology (12%), endodontics (5%), orthodontics (9%), restorative dentistry (2%), pedodontics (2%), and periodontology (1%). Complaints were most commonly filed against private institutions (87%), followed by public institutions (8%) and universities (5%). General dentists were implicated in 93% of the cases, while specialists were involved in 7%. Notably, 8% of the claims involved allegations of negligence on the part of dental laboratories or technicians in the provision of dental prostheses (Table 1).

**Table 1:** General information about the cases

		*n*	%
*Classification of dental treatment*	Oral Surgery	24	24
	Prosthodontics	31	31
	Implantology	12	12
	Oral Diagnosis	14	14
	Endodontics	5	5
	Orthodontics	9	9
	Restorative Dentistry	2	2
	Pedodontics	2	2
	Periodontology	1	1
*Type of providers*	University	5	5
	Private	87	87
	Public	8	8
*Generalist/Specialist*	General Practitioner	93	93
	Specialist	7	7
*Dental laboratory/ technician*	Included	8	8
	Non-included	92	92
*Court decision*	Malpractice	30	30
	Complication	12	12
	Undetermined	29	29
	Unresolved	29	29
*Expert witness*	Yes	59	59
	No	41	41
*Settlement*	Yes	1	1
	No	99	99
	***Total***	100	100

The most frequent reason for malpractice lawsuits was incorrect treatment, accounting for 88% of cases, followed by incomplete treatment (33%), misdiagnosis (32%), patient fault (21%), treatment delays (19%), lack of follow-up (16%), failure to obtain patient consent (10%), delays in diagnosis (3%), document forgery (3%), and infectious disease (2%) (Table 2). A statistically significant difference was observed in the rate of misdiagnosis among the four groups (*p* = 0.020; *p* < 0.05). The rate of misdiagnosis was significantly lower in Group III (13.8%) compared to Groups I (46.7%) and IV (41.4%) (*p* < 0.05), but no significant difference was found between Group III and Group II (*p* > 0.05). There were no significant differences in other litigation reasons across the groups (*p* > 0.05).

**Table 2:** Evaluation of reasons for malpractice litigation and clinical findings based on court decision

	Total	**Malpractice**	**Complication**	Undetermined	Unresolved	p
		**(n = 30)**	(n = 12)	(n = 29)	(n = 29)	
	**(%)**	**n (%)**	**n (%)**	**n (%)**	**n (%)**	
***Reasons***						
Misdiagnosis	%32	14 (%46,7)	2 (%16,7)	4 (%13,8)	12 (%41,4)	0,020*
Incorrect Treatment	%88	29 (%96,7)	10 (%83,3)	23 (%79,3)	26 (%89,7)	^+^0,210
Incomplete Treatment	%33	10 (%33,3)	1 (%8,3)	12 (%41,4)	10 (%34,5)	0,235
Diagnosis Delay	%3	1 (%3,3)	0 (%0)	0 (%0)	2 (%6,9)	^+^0,425
Treatment Delay	%19	6 (%20)	1 (%8,3)	5 (%17,2)	7 (%24,1)	0,691
Lack of Consent	%10	4 (%13,3)	2 (%16,7)	2 (%6,9)	2 (%6,9)	^+^0,663
Neglect	%16	6 (%20)	0 (%0)	8 (%27,6)	2 (%6,9)	^+^0,062
Infectious Diseases	%2	0 (%0)	0 (%0)	1 (%3,4)	1 (%3,4)	^+^0,687
Falsification of Records	%3	3 (%10)	0 (%0)	0 (%0)	0 (%0)	^+^0,065
***Clinical Findings***						
Patient’s Fault	%21	5 (%16,7)	4 (%33,3)	5 (%17,2)	7 (%24,1)	0,602
Discomfort-Pain	%90	29 (%96,7)	11 (%91,7)	25 (%86,2)	25 (%86,2)	^+^0,485
Aesthetic Damage	%47	14 (%46,7)	6 (%50)	11 (%37,9)	16 (%55,2)	0,619
Tooth Damage/ Extraction	%44	17 (%56,7)	6 (%50)	6 (%20,7)	15 (%51,7)	0,026*
Wrong Tooth Removal	%7	3 (%10)	0 (%0)	3 (%10,3)	1 (%3,4)	^+^0,498
Damage to Periodontal Structures	%21	6 (%20)	1 (%8,3)	7 (%24,1)	7 (%24,1)	0,677
Root Injuries	%7	3 (%10)	0 (%0)	1 (%3,4)	3 (%10,3)	^+^0,498
Bleeding	%6	2 (%6,7)	1 (%8,3)	2 (%6,9)	1 (%3,4)	^+^0,915
Prosthetic works with uncleanable part	%1	1 (%3,3)	0 (%0)	0 (%0)	0 (%0)	^+^0,502
TMJ Problems	%9	0 (%0)	0 (%0)	3 (%10,3)	6 (%20,7)	^+^0,029*
Salivary Gland Disorders	%1	0 (%0)	0 (%0)	1 (%3,4)	0 (%0)	^+^0,480
Alveolar bone injury	%13	6 (%20)	1 (%8,3)	3 (%10,3)	3 (%10,3)	^+^0,595
Nerve Damage	%10	3 (%10)	2 (%16,7)	2 (%6,9)	3 (%10,3)	^+^0,824
Surgery Repetition	%18	5 (%16,7)	2 (%16,7)	7 (%24,1)	4 (%13,8)	0,768
New Operation Needed	%5	1 (%3,3)	0 (%0)	2 (%6,9)	2 (%6,9)	^+^0,742
Facial Paralysis	%2	0 (%0)	1 (%8,3)	0 (%0)	1 (%3,4)	^+^0,265
Permanent Paralysis	%1	1 (%3,3)	0 (%0)	0 (%0)	0 (%0)	^+^0,502
Septic Shock	%1	1 (%3,3)	0 (%0)	0 (%0)	0 (%0)	^+^0,502
Malign Hypothermia	%1	1 (%3,3)	0 (%0)	0 (%0)	0 (%0)	^+^0,502
SIRS Triggering	%1	0 (%0)	1 (%8,3)	0 (%0)	0 (%0)	^+^0,060
Vision Loss	%3	0 (%0)	2 (%16,7)	1 (%3,4)	0 (%0)	^+^0,023
Swelling	%3	0 (%0)	2 (%16,7)	1 (%3,4)	0 (%0)	^+^0,023
Brain Edema	%2	0 (%0)	0 (%0)	2 (%6,9)	0 (%0)	^+^0,172
Death	%1	1 (%3,3)	0 (%0)	0 (%0)	0 (%0)	^+^0,502
Other	%2	3 (%10)	1 (%8,3)	9 (%31)	9 (%31)	0,086

Chi-square test + Fisher-Freeman-Halton Exact Test *p < 0.05

Additionally, the most common symptoms reported were discomfort and pain (90%), aesthetic damage (47%), tooth damage or extraction (44%), periodontal tissue damage (21%), need for repeat surgery (18%), postsurgical bone complications (13%), nerve damage (10%), temporomandibular joint (TMJ) disorders (9%), and incorrect tooth removal (7%). Other symptoms included endodontic problems (7%), bleeding (6%), unplanned additional surgery (5%), vision loss or edema (3%), facial paralysis (2%), brain edema (2%), and more rare conditions such as septic shock and death (1%) (Table 2). A statistically significant difference was observed in the rate of tooth damage or extraction (*p* = 0.026; *p* < 0.05), with the undetermined group showing a significantly lower rate (20.7%) compared to the malpractice (56.7%) and unresolved groups (51.7%) (*p* < 0.05), but no significant difference was found when compared to the complications group (*p* > 0.05). The rate of TMJ disorders was also significantly higher in Group IV (20.7%) compared to Groups I and II (0%) (*p* < 0.05), but no significant difference was found between Group IV and Group III (*p* > 0.05). No statistically significant differences were found in the rates of other symptoms across the four groups (*p* > 0.05).

In the cases related to oral surgery, the most common clinical issues were inadequate diagnosis led to unnecessary tooth extraction (25%), complications following third molar extraction (20.8%), nerve injury (16.7%), alveolitis (12.5%), jaw fracture (8,3%) and one case each (4,2%) involved postoperative infection, sinus infections, oroantral injury and cyst recurrence. Other complications were observed in 50% of cases.

Of the 24% of cases related to surgical procedures, 25% were classified as malpractice, 25% as complications, 33.3% as undetermined, and 16.7% as unresolved. A statistically significant difference was observed in the rate of complications following third molar extraction (*p* = 0.001; *p* < 0.05), with the complication group showing a significantly higher rate (*p* < 0.05) compared to the malpractice and undetermined groups, but no significant difference was found between the complication and unresolved groups (*p* > 0.05). No significant differences were observed in the rates of other surgical complications across the four groups (*p* > 0.05) (Table 3).

**Table 3:** Evaluation of clinical findings in surgical procedures based on court decision (*n* = 24)

Clinical findings	Total	Malpractice	Complication	Undetermined	Unresolved	**p**
		(*n* = 11)	(*n* = 1)	(*n* = 12)	(*n* = 7)	
	**(%)**	**n (%)**	**n (%)**	**n (%)**	**n (%)**	
Nerve Injury	%16,7	1 (%16,7)	2 (%33,3)	0 (%0)	1 (%25)	0,365
Jaw Fracture	%8,3	1 (%16,7)	1 (%16,7)	0 (%0)	0 (%0)	0,652
Postoperative Infection	%4,2	0 (%0)	0 (%0)	1 (%12,5)	0 (%0)	1,000
Maxillary Sinus Infection	%4,2	1 (%16,7)	0 (%0)	0 (%0)	0 (%0)	0,667
Alveolitis	%12,5	1 (%16,7)	2 (%33,3)	0 (%0)	0 (%0)	0,257
Oroantral Damage	%4,2	1 (%16,7)	0 (%0)	0 (%0)	0 (%0)	0,667
Leaving the root/ partial removal of impacted third molar	%20,8	0 (%0)	4 (%66,7)	0 (%0)	1 (%25)	0,008*
Cyst Recurrence Surgery	%4,2	0 (%0)	1 (%16,7)	0 (%0)	0 (%0)	0,667
Unnecessary Tooth Extraction	%25	2 (%33,3)	1 (%16,7)	1 (%12,5)	2 (%50)	0,570
Other	%50	3 (%50)	2 (%33,3)	6 (%75)	1 (%25)	0,389

Fisher Freeman Halton Exact Test *p < 0.05

In prosthodontic treatment-related cases, the primary issues included faulty prostheses (48.4%), injuries result in tooth extraction (29%), and chewing discomfort (16.1%), with no statistically significant differences observed across the groups (*p* > 0.05). Other complaints included faulty tooth preparation (16.1%), occlusion problems (16.1%), mouth sores (12.9%), and headaches (12.9%). Less common issues included complications related to denture trauma and unstable dentures (6.5%). Issues such as halitosis, poor-fitting prostheses and negligent injury were reported in 3.2% of cases. The distribution of case outcomes for prosthodontic claims was as follows: 35.5% resulted in malpractice, 3.2% in complications, 38.7% were undetermined, and 22.2% remained unresolved, with no significant differences in the rates of clinical findings across the groups (*p* > 0.05) (Table 4).

**Table 4:** Evaluation of clinical findings in prosthodontic cases based on court decision (*n* = 31)

Clinical findings	Total	Malpractice	Complication	Undetermined	Unresolved	**p**
		(*n* = 11)	(*n* = 1)	(*n* = 12)	(*n* = 7)	
	**(%)**	**n (%)**	**n (%)**	**n (%)**	**n (%)**	
Chewing Discomfort	%25,8	3 (%27,3)	0 (%0)	1 (%8,3)	4 (%57,1)	0,094
Denture Trauma	%6,5	1 (%9,1)	0 (%0)	0 (%0)	1 (%14,3)	0,535
Oral Sores	%12,9	2 (%18,2)	0 (%0)	1 (%8,3)	1 (%14,3)	0,838
Halitosis	%3,2	0 (%0)	0 (%0)	1 (%8,3)	0 (%0)	1,000
Unstable Denture	%6,5	1 (%9,1)	0 (%0)	1 (%8,3)	0 (%0)	1,000
Faulty Preparation of Tooth	%16,1	3 (%27,3)	0 (%0)	1 (%8,3)	1 (%14,3)	0,661
Headache Problem	%12,9	0 (%0)	1 (%100)	2 (%16,7)	1 (%14,3)	0,093
Occlusion Problem	%16,1	2 (%18,2)	0 (%0)	0 (%0)	3 (%42,9)	0,064
Injury Related Tooth Extraction	%29	3 (%27,3)	0 (%0)	3 (%25)	3 (%42,9)	0,839
Poor Fitting Prosthesis	%3,2	1 (%9,1)	0 (%0)	0 (%0)	0 (%0)	0,613
Faulty Prosthesis	%48,4	5 (%45,5)	0 (%0)	5 (%41,7)	5 (%71,4)	0,497
Injury Due to Negligence	%3,2	0 (%0)	0 (%0)	0 (%0)	1 (%14,3)	0,258
Other	%41,9	5 (%45,5)	0 (%0)	7 (%58,3)	1 (%14,3)	0,218

Fisher Freeman Halton Exact Test *p < 0.05

In implantology-related cases, the primary issue observed in all cases was implant failure (100%), followed by periimplantitis (58.3%), bone necrosis (16.7%), and postoperative infections (16.7%). Additionally, one case each involved alveolitis, complications following tooth extraction, and insufficient implant support. The distribution of case outcomes for implantology claims was as follows: 25% resulted in malpractice, 16.7% in complications33.3% were undetermined, and 25% remained unresolved, with no significant differences in the rates of clinical findings across the groups (*p* > 0.05) (Table 5).

**Table 5:** Evaluation of clinical findings in implantology cases based on court decision (*n* = 12)

Clinical findings	Total	Malpractice	Complication	Undetermined	Unresolved	**p**
		(*n* = 3)	(*n* = 2)	(*n* = 4)	(*n* = 3)	
	**(%)**	**n (%)**	**n (%)**	**n (%)**	**n (%)**	
Implant Failure	%100	3 (%100)	2 (%100)	4 (%100)	3 (%100)	-
Periimplantitis	%58,3	0 (%0)	2 (%100)	3 (%75)	2 (%66,7)	0,189
Bone Necrosis	%16,7	0 (%0)	0 (%0)	1 (%25)	1 (%33,3)	1,000
Alveolitis	%8,3	0 (%0)	1 (%50)	0 (%0)	0 (%0)	0,167
Leaving the root/ partial removal of impacted third molar	%8,3	0 (%0)	1 (%50)	0 (%0)	0 (%0)	0,167
Postoperative Infection	%16,7	0 (%0)	1 (%50)	0 (%0)	1 (%33,3)	0,379
Injury Related Tooth Extraction	%8,3	1 (%33,3)	0 (%0)	0 (%0)	0 (%0)	0,667
Insufficient Implant Support	%8,3	0 (%0)	0 (%0)	0 (%0)	1 (%33,3)	0,667

Fisher Freeman Halton Exact Test *p < 0.05

Endodontic-related claims constituted 5% of the total cases, with 20% resulting in malpractice and 80% remaining unresolved. The issues in these cases included incomplete endodontic filling (60%), endodontic overfilling (20%), communication failures (20%), periapical cysts, fistula formation, flare-ups, and complications requiring tooth extraction (20% each). Due to the small number of malpractice cases (one), no statistical comparisons were performed for these claims (Table 6).

**Table 6:** Case details regarding endodontic treatments (*n* = 5)

		*n*	%
***Court Decsion***			
	Malpractice	1	20
	Unresolved	4	80
***Clinical Findings***			
	Incomplete Endodontic Treatment	0 (%0)	3 (%75)
	Overfilling	1 (%100)	0 (%0)
	Patient-Doctor Harmony	0 (%0)	1 (%25)
	Cystic formation	0 (%0)	1 (%25)
	Fistula formation	0 (%0)	1 (%25)
	Flare-up	0 (%0)	1 (%25)
	Tooth extraction	1 (%100)	0 (%0)

In pedodontics-related claims, pain was the predominant complaint, while a malpractice ruling was issued in a case involving tooth extraction or damage. In periodontal treatment-related cases, complaints were for pain and periodontal damage. In restorative dental treatment-related claims, pain was also frequently reported; however, a complication ruling was made in a case involving tooth extraction or damage. In orthodontic treatment-related claims, the primary complaints were aesthetic damage and pain. A complication ruling was issued in a case requiring reoperation, while a malpractice decision was made for a case involving aesthetic damage. In oral diagnosis-related cases, lawsuits were primarily filed due to incorrect or delayed diagnoses, with malpractice rulings issued in 43% of the cases. Due to the small number of malpractice cases, no statistical comparisons were conducted for these claims.

The compensation requested in these cases ranged from 15 TL to 270,000 TL, with a mean of 31,100.68 ± 51,870 TL and a median of 15,000 TL. The compensation awarded ranged from 5 TL to 322,036 TL, with a mean of 30,541.65 ± 76,156.14 TL and a median of 9,955 TL.

## Discussion

The incidence of medical malpractice claims has notably increased in recent years, with variations across countries, and a similar upward trend observed in Türkiye [16, 17]. In our study, the Supreme Court records of cases related to medical malpractice were accessible as of 2000, and the number of cases had increased as of 2012 and these findings similarly reflect the results obtained by studies in the literature. Such increases are likely to reflect growing public awareness, media attention, and evolving societal expectations regarding healthcare standards [17]. One significant factor contributing to this rise is heightened societal sensitivity to medical errors, amplified by media portrayals of malpractice cases. These portrayals, often fueled by misinformation, can lead to patient distrust and encourage legal action. In this context, patients may be more inclined to pursue legal recourse, particularly in the absence of clear, reliable dialogue regarding treatment risks and outcomes [17, 18]. Distinguishing between medical malpractice and complications remains a challenging task, with international literature indicating that medical malpractice cases do not overlap with cases involving clinical errors [19]. In the same vein, the Harvard Medical Practice study reported that only 8 out of 280 patients who experienced adverse events due to clinical errors filed malpractice lawsuits. The study concluded that there was a clear overestimation, with the majority of malpractice lawsuits failing to meet the criteria for adverse events resulting from clinical errors [19]. In our study, 30% of the cases were classified as malpractice, which is higher than some reports in the literature. This discrepancy likely stems from the inherent difficulty in distinguishing between malpractice, complications, and expected patient outcomes.

Furthermore, as Hojat et al. [20] observed, factors such as physician empathy and communication play a crucial role in reducing complaints, with female physicians, in particular, receiving fewer complaints due to their more empathetic interactions with patients. In our study, since gender information was not available in the Supreme Court decisions, it was not possible to be determined as an evaluation criterion. Furthermore, gender-related data were unavailable in our study, limiting our ability to explore this potential factor.

In our cohort, 93% of complaints were directed at general practitioners, while only 7% involved specialists. This is consistent with findings from previous studies, which reported that the majority of malpractice cases across various countries were attributed to general dentists [15, 21–23]. Additionally, 87% of complaints in our study were lodged against private institutions, a trend also observed in the research on dental malpractice by Abomalik et al. [21]. The prevalence of complaints against private dental practices may be linked to higher patient expectations and a greater emphasis on aesthetic outcomes, especially in procedures such as prosthodontics. However, believing that medical negligence or errors are rarely seen in public health units may not reflect the reality, as dentists often work in private practices, which can limit any comprehensive understanding of risk management related to dental care, good documentation and adherence to informed consent protocols as stated similarly at a recent study in 2024 by Wu et al. [14]. Informed consent is a critical aspect of medical and dental practice, yet this principle remains an area of concern [17, 24]. In our study, 10% of the cases involved a lack of informed consent, underscoring the importance of clear communication with patients pertaining to the risks and benefits of specific treatments. Challenges inherent in the doctor-patient relationship—often exacerbated by asymmetries in knowledge and authority—may contribute to the persistence of complaints, especially when patient expectations go unmet. The 2005 study by Ozdemir on malpractice claims in Turkey found that 25% of dentists were deemed guilty not for the treatment they provided, but for failing to obtain informed consent [17]. Despite an upward trend observed in Turkey, the findings of the current study support the notion that failures in communication and the establishment of trust between patients and healthcare providers can lead to dissatisfaction and, consequently, legal action.

In line with earlier studies [15, 22, 23, 25], prosthodontics and oral surgery were the most frequent sources of dental malpractice claims in our study. Alsaeed et al. [15], Kiani and Azadi [26], and Montagna et al. [27] reported that the majority of claims were related to prosthodontic litigations, whereas Fernandez et al. [28] and Moles et al. [29] found that oral surgery was associated with the highest number of claims. The present study found that complaints were most commonly associated with prosthodontic treatments and oral surgeries in agreement with previous literature; however, complaints related to prosthodontic treatments were primarily driven by issues related to inadequate function and patient comfort, rather than aesthetic concerns alone. This highlights the complex nature of prosthodontic care, where high patient expectations and the involvement of dental technicians may contribute to dissatisfaction [14, 30]. Similarly, oral surgical procedures, particularly misdiagnosis led to unnecessary teeth extractions and complications following third molar extractions, were a significant source of complaints in our study. The surgical removal of third molars, typically performed under local anesthesia, carries risks such as pain, swelling, and trismus, which generally resolve with appropriate care, while less common complications, including damage to adjacent teeth, infections, and alveolitis, may also arise, with site-specific issues such as oroantral communication and trigeminal nerve injuries [24, 31, 32]. The most prevalent complications in this group were incomplete removal or partial extraction of impacted third molars, followed by nerve injury and alveolitis, consistent with the existing literature [31–33]. Infections, both early and late postoperative, were frequent causes of litigation in the cases examined in our study, consistent with previous reports [32, 33], underscoring the importance of stringent infection prevention and control practices in dental surgery. The experience level of the dentist plays a crucial role in achieving outcomes within the range of reported complication rates [32]. Some studies have shown that factors such as prolonged surgery, impaction type, and complex procedures like osteotomy have a significant impact on post-extraction complications, including uncontrolled pain and alveolitis [31, 32]. However, in our study, however, data regarding the dentists’ experience, patient age, extraction type, or procedure time were not available in the Supreme Court decisions, making it impossible to use these factors as evaluation criteria. As a result, we were unable to explore these potential influences. In addition, de Abreu et al. emphasized that personalized preoperative and perioperative risk assessments, along with the implementation of effective risk management strategies, are critical for ensuring optimal care and minimizing legal risks [31]. The causes of litigation identified in our study point to the need for a more comprehensive analysis and enhanced communication with patients prior to oral surgery, especially when deciding whether to extract or preserve the natural tooth as, well as addressing site-specific complications such as oroantral communications in upper third molars and trigeminal nerve injuries in lower molar extractions.

Implant dentistry has become a cornerstone of modern dental practice, offering evidence-based solutions for rehabilitating edentulous patients. Advances in clinical techniques and technology have expanded implant therapy from a specialist-only procedure to a common treatment in general practices, increasing the responsibility of all clinicians involved in implant dentistry to provide care at appropriate standards [34]. In the current study, implantology ranks third in judicial processes, following prosthodontics and oral surgery related cases. The results of the present study are somewhat consistent with those of Kim’s research, which reviewed medicolegal issues in South Korea between 2016 and 2023, identifying dental implant placement as the most common dental procedure associated with adverse events [35]. Additionally, the research conducted by Corto-Real et al. provided Portuguese data on professional liability in dentistry, categorizing iatrogenic sequelae into risks and malpractice, with implantology identified as one of the primary areas of concern [5]. The study by Pinchi et al. [36], which examined malpractice claims in implant dentistry in Italy between 2006 and 2010, found that 90.1% of patients required implant replacement, with periimplantitis observed in 30.6% of cases. Additionally, the study revealed that more than a quarter of cases required alterations to the original prosthetic design due to implant loss, while alveolar bone loss necessitating regenerative techniques or bone grafts was noted in over half of the implant failure cases [36]. Similarly, our study identified implant failure as the most common issue, followed by periimplantitis, with other complications such as bone necrosis contributing to implant failure. Overall, these findings underlined the multifaceted nature of implantology, emphasizing the importance of comprehensive patient evaluation, surgical precision, and meticulous post-treatment care in minimizing the risk of malpractice and ensuring successful clinical outcomes.

Rising compensation payouts being awarded in malpractice cases is another significant trend. Zentz reported that more than 1,500 dental malpractice claims were filed annually in the National Practitioner Data Bank (NPDB) dataset between September 1990 and December 2018 [12]. Notably, the average malpractice payment for dentists reported to the NPDB in 1991 was $23,178. When adjusted for inflation, this amount is approximately $49,800 in 2022. However, in 2022, the average malpractice payment for dentists had risen to $113,188, representing an increase of approximately 2.25 times compared to 1991 [12].

The study conducted by Vadde et al. analyzed dental malpractice claims in India from 2018 to 2022, reporting that plaintiffs received compensation ranging from Rs. 50,000 to Rs. 500,000 [22]. Similarly, Kim’s [35] investigation of malpractice claims in South Korea between 2016 and 2023 found the average claimed amount to be USD 71,120 ± 166,369 (KRW 92,456,328 ± 218,879,276), with an average awarded compensation of USD 11,506 ± 8,165 (KRW 14,958,121 ± 10,614,527). In cases involving psychological damage, the average awarded compensation was USD 4,692 ± 2,614 (KRW 6,100,000 ± 3,397,941) [35].

In Spain, Bordonaba-Leiva et al. [24] analyzed oral and maxillofacial surgery malpractice claims from 1990 to 2014, finding an average compensation of €19,639.58 for cases involving professional liability. The mean compensation awarded in out-of-court settlements and court-resolved procedures was €13,318.39 and €24,217, respectively [24].

In Saudi Arabia, Alsaeed et al. [22] examined dental malpractice cases from 2017 to 2020, reporting that the average indemnity paid by defendants to plaintiffs was SAR 26,297 ± 44,739, with institutions paying SAR 23,356 ± 34,622. The average financial penalty paid by defendants to the government was SAR 8,907 ± 13,750, with institutions paying SAR 34,375 ± 30,066. Additionally, the average compensation for reconciliation provided to plaintiffs was SAR 14,761 ± 20,859 [22].

No information about compensation rates has been found in the literature conducted in Türkiye. In our study, compensation claims ranged from 15 TL to 270,000 TL, with the highest awarded compensation reaching 322,036 TL. This mirrors trends observed in other healthcare sectors, where the financial burden of malpractice litigation is substantial [37, 38]. The escalation in compensation payouts reinforces the need for comprehensive professional liability insurance and a more transparent system for managing malpractice claims. Additionally, the implementation of a national electronic reporting system for dental malpractice cases could improve data accuracy and facilitate more effective decision-making in legal proceedings.

This 23-year litigation analysis reveals that approximately 70% of dental professionals involved in malpractice lawsuits are not found liable. The limitations of the current study include the inability to assess the complexity of the cases and the variability of anatomical structures, as well as factors such as the patient’s medical history and lifestyle habits, including smoking, alcohol consumption, and bruxism, all of which are known to contribute to post-treatment complications. Furthermore, the dentist’s experience and the technical expertise of the dental laboratory may influence treatment outcomes. Lastly, the relatively small sample size derived from the Supreme Court of Turkey, along with challenges in accessing historical case data, may limit the generalizability of the findings.

## Conclusion

In conclusion, this study underscores the critical importance of thorough planning, detailed assessments, and preventive measures in dental practice, particularly within the fields of prosthodontics, oral surgery, and implantology, as all three specialties involve characterized by invasive procedures, extended treatment durations, relatively high costs, and frequent post-treatment discomfort, are inherently linked to higher patient dissatisfaction and an increased likelihood of malpractice lawsuits.

Despite international guidelines and recommendations, there is a need to strengthen oversight of complex procedures to ensure their proper execution, as well as to guide clinicians in patient selection and decision-making. This would help clinicians assess whether they possess the necessary skills for a given treatment or if a referral is required, enabling them to build experience gradually. Additionally, this approach would play a critical role in education, contributing to the development of training programs that may help reduce the incidence of legal disputes in the future. Future research should focus on larger sample sizes and explore factors that influence legal liability in dental malpractice cases, including the economic and social implications of malpractice litigation.

## Data Availability

All cases were obtained from the Supreme Court of Appeals IT Directorate database (karararama.yargitay.gov.tr) using the keywords “Malpractice in Dentistry (in Turkish: Diş Hekimliğinde Malpraktis)” and “Malpractice Cases in Dentistry (in Turkish: Malpraktis Davaları Diş Hekimliği)”.
